# Perinatal outcomes after laser photocoagulation of anastomoses in monochorionic pregnancies

**DOI:** 10.1590/1806-9282.20250330

**Published:** 2025-09-19

**Authors:** Talita Micheletti, Gabriela Pigozzi, Marina Pereira Domingues, Mariana Ziliotto Sgnaolin, Marcelo Brandão da Silva, Mauricio Obal Colvero, Edward Araujo

**Affiliations:** 1 Santa Casa de Porto Alegre, Instituto Materno-Fetal Celso Rigo, Fetal Medicine Unit – Porto Alegre (RS), Brazil.; 2 Universidade Federal de Ciências da Saúde de Porto Alegre, Department of Obstetrics and Gynecology – Porto Alegre (RS), Brazil.; 3 Universidade Federal de São Paulo, Escola Paulista de Medicina, Department of Obstetrics – São Paulo (SP), Brazil.; 4 Universidade Municipal de São Caetano do Sul, Discipline of Woman Health, São Caetano do Sul (SP), Brazil.

**Keywords:** Twin pregnancy, Twin-to-twin transfusion syndrome, Fetal therapies

## Abstract

**OBJECTIVE:**

To describe the perinatal outcomes of monochorionic pregnancies complicated with twin-to-twin transfusion syndrome, laser photocoagulation of placental anastomoses was presented as an initial experience in a reference center in South Brazil.

**METHODS:**

Case series between October 2020 and March 2024, including monochorionic twin/triplet pregnancies complicated with twin-to-twin transfusion syndrome. This syndrome was classified according to Quintero's stage. The maternal and surgical characteristics, as well as the perinatal outcomes were assessed.

**RESULTS:**

Of the 17 pregnant women diagnosed with twin-to-twin transfusion syndrome, 11 (64.7%) were treated at our center, including 9 monochorionic-diamniotic, 1 dichorionic-triamniotic, and 1 monochorionic-diamniotic triplet. Of the 9 laser ablations of placental anastomoses included in the study, 8 (88.9%) had 2 or more live births, and 1 (11.1%) had no live births. The mean gestational age at surgery was 21.9 weeks. Regarding Quintero stage at the time of surgery, 2 were stage I, 3 were stage II, and 4 were stage IV. The interval between surgery and delivery was 10.8 weeks, with a mean gestational age at delivery of 32.7 weeks. The mean birth weight of the recipient was 1,845 g, and that of the donor was 1,667.8 g.

**CONCLUSION:**

Our initial experience with laser ablation of placental anastomoses in monochorionic pregnancies complicated by twin-to-twin transfusion syndrome showed good perinatal outcomes with lower complication rates.

## INTRODUCTION

Twin-to-twin transfusion syndrome (TTTS) complicates approximately 10–15% of monochorionic pregnancies, usually manifesting between 16 and 26 weeks of gestation. It is caused by an imbalance in the exchange of blood that normally occurs between the two fetuses in a monochorionic placenta through existing vascular connections^1^. Placental vascular anastomoses can be of three types: arterio-arterial, veno-venous, or arterio-venous (AV). AV are the anastomoses that have the potential to cause an imbalance in the intertwin circulation, receiving an arterial supply from one fetus, called the donor, and providing a venous drainage to the other fetus, called the recipient^2^. In practice, hypervolemia, polyuria, and polyhydramnios are observed in the recipient fetus, and hypovolemia, oliguria, and oligohydramnios in the donor fetus^3^.

TTTS is a serious condition that if left untreated, has a pregnancy loss rate as high as 100% if diagnosed before 20 weeks and more than 80% between 21 and 26 weeks. In addition, severe sequela occurs in up to 80% of survivors^4^, indicating the need for fetal intervention. The therapeutic options available for the management of TTTS include amniodrainage (serial removal of amniotic fluid), septostomy (intentional creation of a perforation in the intertwin membrane), and laser ablation of the placental vessels^5^, the latter being the current treatment of choice regardless of staging^1,4^.

Evidence of the superiority of treatment with laser ablation of the placental vessels came from a randomized, multicenter trial conducted in 2004 that compared perinatal outcomes in pregnancies diagnosed with severe TTTS treated with either amniodrainage or laser ablation of the placental vessels for up to 26 weeks^6^. Laser ablation of the placental vessels is the only procedure that acts on the physiopathology of TTTS^7^. Although the severity of TTTS is greater at Quintero stage II and above^8^, some centers recommend performing laser ablation of the placental vessels at stage I and above because of the risk of unpredictable progression and fetal death in the event of expectant management^1,4^.

The aim of this study was to present the first experience with laser ablation of the placental vessels in monochorionic pregnancies with TTTS in a reference center in South Brazil.

## METHODS

Case series with data of all monochorionic twin pregnancies diagnosed with TTTS that underwent laser ablation of placental anastomoses at the Instituto Materno Fetal Celso Rigo of Santa Casa de Porto Alegre, Rio Grande do Sul, Brazil, from October 2020 to March 2024. This study was approved by the Ethics Committee of the Santa Casa de Porto Alegre (CAAE. 46398821.6.0000.5335) and the patients signed informed consent.

The initial evaluation of twin complications was performed by the senior surgeon and team following a standard protocol^4^, and the pregnancies diagnosed with TTTS were staged according to Quintero classification^9^.

**Figure 1:**
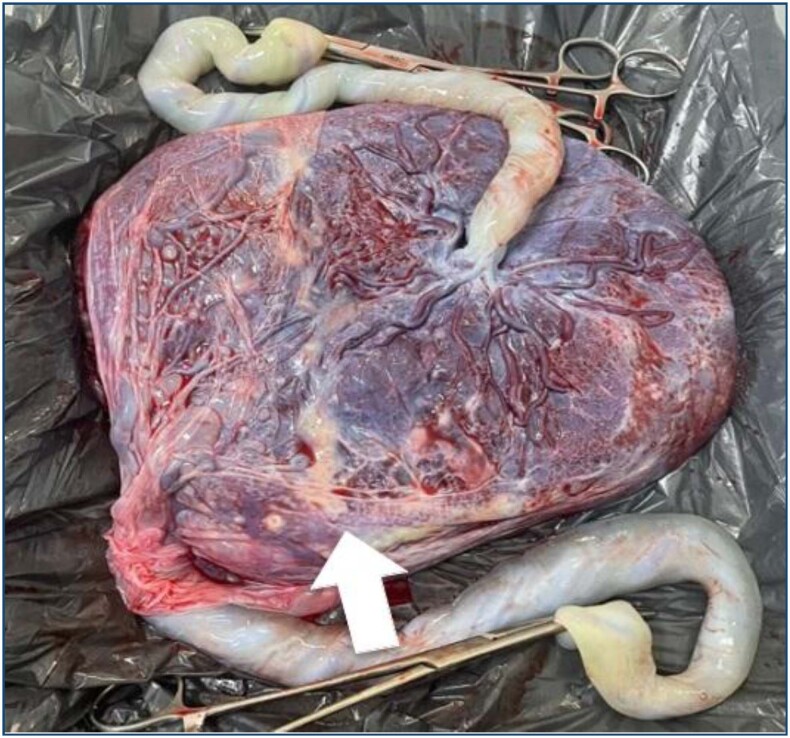
Gross pathology of the placenta after delivery in a monochorionic twin pregnancy submitted to laser ablation of the anastomoses using the Solomon technique. Note the "coagulation line" (arrow) drawn from one side of the placenta to the other.

The surgeries were performed according to the service's standard fetoscopic technique, under loco-regional anesthesia with sedation, using a single entry through a 10Fr cannula (direct puncture with a trocar) guided by ultrasound and Doppler to avoid maternal and placental vessels. A 2-mm straight fetoscope was then introduced, with the diode laser fiber inserted into the working channel of the fetoscope. Laser coagulation was performed at 30 W power, using selective, mixed (selective with some "stripes" of continuous coagulation), or Solomon techniques (continuous coagulation line) (Figure 1). The choice of technique was made by the lead surgeon depending on the characteristics of the vascular equator (placental vessel communications). At the end of the procedure and after removing the fetoscope, amniodrainage was performed until the largest vertical pocket without polyhydramnios was achieved, followed by removal of the cannula under ultrasound guidance. The patients remained hospitalized for approximately 24–48 h, according to protocol, and were discharged for follow-up at their original center or at the Santa Casa Fetal Medicine service, if that was their choice.

The data collected were entered into an Excel for MAC 2024 spreadsheet (Microsoft Corp., Redmond, WA, USA) that included the following information: time interval from indication to surgery, gestational age (GA) at surgery, TTTS stage (according to Quintero's classification^9^) and stage progression before surgery, laser coagulation technique, duration of the surgery, time between surgery and perinatal outcome, presence of complications, GA at delivery, birth weight, and presence or absence of fetal or neonatal death.

Descriptive statistics were analyzed using STATA version 14.0 (Stata Corp., College Station, TX, USA), with results presented in absolute numbers (percentages) or mean (±standard deviation).

## RESULTS

During the period analyzed, 45 patients with monochorionic twin pregnancies were referred to the service for suspected complications. Of these pregnant women, 4 (9%) presented with the death of one or both fetuses, one due to stage V TTTS, one due to selective fetal growth restriction (FGR) with recent death, one due to twin-reversed arterial perfusion (TRAP) sequence, and one with no defined etiology. Of the 41 pregnant women with all fetuses alive, 17 (41.4%) had criteria for TTTS, 14 (34.1%) for selective FGR, 6 (14.6%) had discordant fetal malformations, 1 (2.4%) was diagnosed with twin anemia-polycythemia sequence (TAPS), and 3 (7.3%) had other diagnoses.

Of the 17 pregnant women diagnosed with TTTS, 11 (64.7%) were treated at our center, including 9 monochorionic-diamniotic, 1 dichorionic-triamniotic, and 1 monochorionic-diamniotic triplet. Of these, 10 underwent laser ablation of placental anastomoses without previous interventions. One pregnant woman was excluded from the analysis due to loss to follow-up. Of the 9 laser ablation of placental anastomoses lasers included in the study, 8 (88.9%) had 2 or more live births, and 1 (11.1%) had no live births. The two triplet pregnancies, including one with a pair of monoamniotic twins, progressed with all three fetuses alive. The time interval from initial evaluation and indication to the fetal surgery was 1 day or less in 6 patients (67%) and more than 3 days in the other 3 (33%). From those with delayed surgery, 2 were stage IV and 1 presented progression of Quintero stage from III to IV in this interval.

Regarding characteristics of the patients at the time of the surgery, the mean GA was 21.9 (±1.84) weeks. Regarding the Quintero stage at the time of surgery, 2 pregnant women were in stage I, 3 in stage II, and 4 in stage IV. Laser ablation of placental anastomoses was performed using a selective technique in 4 patients (45%), mixed in 3 (33%), and Solomon in 2 (22%). Mean surgical time was 43.4 (±10.28) min.

In the follow-up, the interval between surgery and delivery was 10.8 (±4.09) weeks, with a mean GA at delivery of 32.7 (±3.7) weeks. Postoperative complications were reported in two pregnant women: one developed mirror syndrome, and another had an unintentional septostomy. None presented intrauterine fetal death.

Concerning perinatal outcomes, the mean birth weight of the recipient was 1,845 (±744) g, and the donor was 1,667.8 (±656) g, including triplet pregnancies; excluding them, the mean birth weights of the recipient and donor were 1,988.4 (±785) and 1,816 (±672) g, respectively. It should be noted that 44% of the pregnant women underwent postoperative follow-up and delivery planning at their services of origin. To date, no mid- or long-term follow-up has been performed. Tables 1 and 2 show the intrauterine surgery characteristics and the perinatal outcomes, respectively.

**Table 1:** Intrauterine surgery characteristics.

Intrauterine surgery characteristics		(n=9)
Gestational age at surgery (weeks) mean (±SD)		21.9 (±1.84)
Quintero's stage		
	I	2 (22.2%)
	II	3 (33.3%)
	III	0 (0)
	IV	4 (44.4%)
Laser coagulation technique		
	Selective	4 (44.4%)
	Mixed	3 (33.3%)
	Solomon	2 (22.2%)
	Duration of the surgery (min) mean (±SD)	43.3 (10.28)

SD: standard deviation.

**Table 2:** Perinatal outcomes.

Perinatal outcomes	(n=9)
Time between surgery and delivery (weeks) mean (±SD)	10.8 (±4.09)
Gestational age at delivery (weeks) mean (±SD)	32.7 (±3.7)
Birth weight of recipient (g) mean (±SD)	1,845 (±744)
Birth weight of donor (g) mean (±SD)	1,667.8 (±656)

SD: standard deviation.

With regard to the other cases of monochorionic diamniotic twin pregnancies treated at the service with complications other than TTTS, the only pregnant woman diagnosed with TAPS underwent intrauterine transfusion at 27.8 weeks and evolved with one live newborn at 37.1 weeks and a birth weight of 2,626 g. The pregnant women with selective FGR, mostly Gratacós types II and III^10^, had a GA at delivery of 31.3 weeks (±5.12), with the adequate newborn weighing 1,846 g (±497) and the restricted newborn weighing 1,350 g (±476).

## DISCUSSION

The benefits of laser ablation of placental anastomoses and its superiority over other therapeutic modalities in cases of TTTS have already been demonstrated in the international literature^5,6^. However, there are few studies on the results of this intrauterine surgery in Brazil^8,11,12^, due to its limited availability in different regions of the country, the high cost of the material, and the lack of specialized professionals, as well as the difficulty pregnant women have in accessing reference centers.

Our initial experience using laser ablation of placental anastomoses in cases of TTTS showed good perinatal outcomes, with 8 (88.9%) having two or more live births. Barbosa et al.^12^ showed their initial experience in a reference center in São Paulo, Brazil, with 24 fetoscopic laser photocoagulations; 11/21 (45.8%) had two live births. Other initial experiences in Brazil demonstrated double twin neonatal survival of 2/5 (40%)^13^, 15/19 (26.3%)^14^, 12/30 (40%)^8^, and 2/10 (20%)^15^. Swiatkowska-Freund et al.^16^ showed their initial experience in a tertiary center in Poland with 91 laser ablations of placental anastomoses obtained 49/71 (57.6%) of two live births. Despite the small number of cases evaluated in our series, the initial results with two live newborns proved superior to national and international studies.

In our casuistic, a complete Solomon technique was used in two cases of TTTS. This technique consists of a line photocoagulation laser in the placental equator to achieve the small AV anastomosis, reducing the risk of residual anastomosis and consequently TTTS recurrence of secondary TAPS. The Solomon technique showed a higher survival rate for both twins (84.6 vs. 46.1%) and a higher overall neonatal survival rate [45/52 (86.5%) vs. 94/152 (61.8%)] than the selective technique, respectively^17^. However, it is associated with an increased risk of placental abruption and preterm labor compared to the selective coagulation technique^18^. Those were some of the reasons why selective or mixed techniques were preferred.

In our study, two pregnant women with Quintero's stage I underwent laser ablation of placental anastomoses. Intrauterine surgery in this stage is controversial because a randomized controlled trial comparing laser to expectant management showed no significant differences in live births and severe neurologic morbidity^19^. However, 60% of cases in stage I will progress and require rapid transfer to a surgical center, justifying the intrauterine surgery.

This article has limitations, first, the small sample size, but it represents the first experience of a tertiary service in South Brazil. Second, despite the small sample size, our results in terms of survival of one or both fetuses were superior to first experiences from other international centers and with larger case numbers. We believe that the use of the Solomon and mixed techniques in the majority of cases may have contributed to the good perinatal outcomes. Our findings showed that the laser photocoagulation of placental anastomoses in TTTS cases is a relatively simple technique with a short learning curve.

## CONCLUSION

In conclusion, our initial experience with laser ablation of placental anastomoses in monochorionic pregnancies complicated by TTTS showed good perinatal outcomes with lower complication rates. Future studies with larger numbers of cases are needed to confirm our initial results.

## Data Availability

The datasets generated and/or analyzed during the current study are available from the corresponding author upon reasonable request.

## References

[1] Bamberg C, Hecher K (2022). Twin-to-twin transfusion syndrome: controversies in the diagnosis and management. Best Pract Res Clin Obstet Gynaecol.

[2] Denbow ML, Cox P, Taylor M, Hammal DM, Fisk NM (2000). Placental angioarchitecture in monochorionic twin pregnancies: relationship to fetal growth, fetofetal transfusion syndrome, and pregnancy outcome. Am J Obstet Gynecol.

[3] Lewi L, Gucciardo L, Mieghem T, Koninck P, Beck V, Medek H (2010). Monochorionic diamniotic twin pregnancies: natural history and risk stratification. Fetal Diagn Ther.

[4] Micheletti T, Eixarch E, Bennasar M, Martinez JM, Gratacos E (2021). Complications of monochorionic diamniotic twins: stepwise approach for early identification, differential diagnosis, and clinical management. Mater Fetal Med.

[5] Slaghekke F, Zhao DP, Middeldorp JM, Klumper FJ, Haak MC, Oepkes D (2016). Antenatal management of twin-twin transfusion syndrome and twin anemia-polycythemia sequence. Expert Rev Hematol.

[6] Senat MV, Deprest J, Boulvain M, Paupe A, Winer N, Ville Y (2004). Endoscopic laser surgery versus serial amnioreduction for severe twin-to-twin transfusion syndrome. N Engl J Med.

[7] Behrendt N, Galan HL (2016). Twin-twin transfusion and laser therapy. Curr Opin Obstet Gynecol.

[8] Peralta CF, Ishikawa LE, Passini R, Bennini JR, Nomura ML, Rosa IRM (2009). [Natural history of monochorionic diamniotic twin pregnancies with and without twin-twin transfusion syndrome]. Rev Bras Ginecol Obstet.

[9] Quintero RA, Morales WJ, Allen MH, Bornick PW, Johnson PK, Kruger M (1999). Staging of twin-twin transfusion syndrome. J Perinatol.

[10] Gratacós E, Lewi L, Muñoz B, Acosta-Rojas R, Hernandez-Andrade E, Martinez JM (2007). A classification system for selective intrauterine growth restriction in monochorionic pregnancies according to umbilical artery Doppler flow in the smaller twin. Ultrasound Obstet Gynecol.

[11] Pedreira DA, Acácio GL, Drummond CL, Oliveira Rde C, Deustch AD, Taborda WG (2005). Laser for the treatment of twin to twin transfusion syndrome. Acta Cir Bras.

[12] Barbosa MM, Martins Santana EF, Milani HJF, Elito J, Araujo E, Moron AF (2018). Fetoscopic laser photocoagulation for twin-to-twin transfusion syndrome treatment: initial experience in tertiary reference center in Brazil. Obstet Gynecol Sci.

[13] Pedreira DA, Acácio GL, Drummond CL, Oliveira Rde C, Deustch AD, Taborda WG (2005). Laser for the treatment of twin to twin transfusion syndrome. Acta Cir Bras.

[14] Ruano R, Brizot Mde L, Liao AW, Zugaib M (2009). Selective fetoscopic laser photocoagulation of superficial placental anastomoses for the treatment of severe twin-twin transfusion syndrome. Clinics (Sao Paulo).

[15] Rezende TMS, Weihermann V, Fachin CG, Bruns RF, Dias AIBS (2021). Twin-twin transfusion syndrome - a University Hospital experience with intrauterine treatment. Rev Col Bras Cir.

[16] Swiatkowska-Freund M, Pankrac Z, Preis K (2012). Results of laser therapy in twin-to-twin transfusion syndrome: our experience. J Matern Fetal Neonatal Med.

[17] Ruano R, Rodo C, Peiro JL, Shamshirsaz AA, Haeri S, Nomura ML (2013). Fetoscopic laser ablation of placental anastomoses in twin-twin transfusion syndrome using ‘Solomon technique’. Ultrasound Obstet Gynecol.

[18] D’Antonio F, Herrera M, Oronzii L, Khalil A (2022). Solomon technique vs selective fetoscopic laser photocoagulation for twin-twin transfusion syndrome: systematic review and meta-analysis of maternal and perinatal outcomes. Ultrasound Obstet Gynecol.

[19] Stirnemann J, Slaghekke F, Khalek N, Winer N, Johnson A, Lewi L (2021). Intrauterine fetoscopic laser surgery versus expectant management in stage 1 twin-to-twin transfusion syndrome: an international randomized trial. Am J Obstet Gynecol.

